# Preventive Care for Adults With Cerebral Palsy and Other Neurodevelopmental Disabilities: Are We Missing the Point?

**DOI:** 10.3389/fnint.2022.866765

**Published:** 2022-04-07

**Authors:** Daniel G. Whitney, Michelle L. Rabideau, Michael McKee, Edward A. Hurvitz

**Affiliations:** ^1^Department of Physical Medicine and Rehabilitation, University of Michigan, Ann Arbor, MI, United States; ^2^Institute for Healthcare Policy and Innovation, University of Michigan, Ann Arbor, MI, United States; ^3^Department of Family Medicine, University of Michigan, Ann Arbor, MI, United States

**Keywords:** primary care, secondary care, cerebral palsy, neurodevelopmental disabilities, aging, preventive care

## Abstract

Preventive care techniques are cornerstones of primary care for people with neurodevelopmental disabilities such as cerebral palsy (CP). However, well-established methods evaluating health constructs may not be applied in the same way for adults with CP, as compared to the general population, due to differences in anatomy/physiology, leading to missed opportunities for interventions, medication modifications, and other primary/secondary prevention goals. One barrier to care prevention comes from misinterpretation of values to capture health constructs, even when measurements are accurate. In this Perspective, we emphasize the need for differential interpretation of values from commonly used clinical measures that assess for well-known medical issues among adults with CP obesity risk, bone health, and kidney health. We provide technical, but simple, evidence to showcase why the underlying assumptions of how some measures relate to the health construct being assessed may not be appropriate for adults with CP, which may apply to other neurodevelopmental conditions across the lifespan.

## Introduction

Many children with CP live well into their adult years creating a growing population of adults with CP (Brooks et al., 2014). Unfortunately, clinical care for adults with CP is fraught with uncertainty. Many screening protocols and other preventive care techniques to assess health constructs (e.g., obesity risk) to inform clinical decision making may not be applied in the same way for adults with CP due to differences in their anatomy and physiology from an array of interacting factors, such as abnormal development, motor dysfunction, low activity, malnutrition, medications, and surgeries, among other factors (Kuperminc et al., 2009; Herrera-Anaya et al., 2016; Oftedal et al., 2016; Whitney et al., 2019b). An inability to accurately capture health constructs in clinically meaningful ways can lead to missed opportunities for primary and secondary prevention goals to reduce the risk for the observed functional loss and premature morbidity and mortality common to CP (Day et al., 2007b; van der Slot et al., 2013; Ryan et al., 2014; Himmelmann and Sundh, 2015; Heyn et al., 2019; O’Connell et al., 2019; Smith et al., 2019; Whitney et al., 2019a, 2021a).

Some screening and preventive measures pose a challenge to perform accurately, if at all, such as a bone density scan among individuals with involuntary movement which can affect image quality. Clinicians may not have the resources or familiarity in how to screen for conditions among adults with cognitive impairments (e.g., communication for mental health screening), restricted mobility (e.g., difficulty standing for mammograms), or wheelchairs (e.g., clinician lacks support to transfer patient to an examination table, such as hoists; McColl et al., 2008; Iezzoni et al., 2021).

An important measurement-level challenge that has garnered little attention to date stems from misinterpretation of values to capture a specific health construct, like the measurement, body mass index (BMI), to assess the health construct, obesity risk. Even when values derived from measurements are precise and accurate, they can still be misleading due to underlying assumptions of how the measurement value (or value range) relates to the construct being assessed, which may not be appropriate for individuals with CP or other neurodevelopmental conditions.

The focus of this Perspective is to highlight the potential need for differential interpretation of values derived from clinical measures for adults with CP. While the population in this Perspective is adults with CP, the concepts discussed may be relevant to children with CP and other neurodevelopmental conditions across the lifespan. We provide examples using common clinical measures that assess for well-known medical issues for adults with CP, namely obesity risk and bone health. We also discuss clinical measures to assess kidney function for adults with CP, which is an emerging priority for clinical care.

While there are many salient health constructs to discuss, such as metabolic syndrome (Heyn et al., 2019), mental health, and spasticity, we use the three examples of obesity risk, bone health, and kidney health given four commonalities relevant to the scope of this work. First, the common clinical measures to assess for these health constructs rely on underlying assumptions from the general population that may not hold true for many adults with CP and are further complicated by the severity of CP. Second, these measures are generally accurate and repeatable, so the discordance in the value capturing the health construct for adults with CP is not necessarily due to measurement error, but rather, human error in correctly interpreting the value within the context of CP. Third, these three health constructs are strongly implicated in the pathogenesis of unhealthful aging for adults with CP. Providing deeper insight into how to better interpret these methods may have a larger clinical impact for adults with CP. For example, it is well known that there is a high burden of obesity-related morbidity and mortality for adults with CP (e.g., cardiometabolic disease; Heyn et al., 2019; Ryan et al., 2019; Whitney et al., 2021a). Recent work has identified fragility fractures as a risk factor for premature cardiorespiratory morbidity and all-cause mortality for adults with CP (Etter et al., 2020; Whitney et al., 2020a, b). Emerging work is documenting just how common kidney disease is and its robust association with mortality for adults with CP (Whitney et al., 2020c, d, 2021b; Whitney and Oliverio, 2021). Fourth, health complications from obesity, poor bone health, and poor kidney function can be prevented, delayed, or at least better managed with early intervention strategies, but these efforts rely on sufficient detection in the clinical setting.

While this Perspective focuses on three health constructs, the thought processes can be applied to other health domains. We encourage individuals to consider how other clinical tests not discussed herein may be misinterpreted in the context of aging with CP due to an alteration in some underlying assumption driving the test’s interpretation.

## Challenging Underlying Assumptions

We begin by introducing a basic overview of aspects of CP that challenge the underlying assumptions derived from the general population when interpreting values from clinical measurements. We refer to “severity” of CP to indicate the severity of the impact of etiological factors on the developmental alterations of anatomy and physiology of the individual, which also allows for transference of concepts to other adult populations with neurodevelopmental disabilities.

Children with CP often experience lags in height and body mass growth which is exacerbated by the severity of CP (Oftedal et al., 2016). Further, the lag in body mass gains in general are greater than the lag in height gains, thus uniquely impacting measurements that include height and body mass (e.g., BMI; Day et al., 2007a). In simple terms, body mass refers to the composition of fat mass and fat-free mass. In the general non-obese population, body mass is primarily composed of fat-free mass. Individuals with CP not only exhibit lower muscle mass (major component of fat-free mass) which is lower with greater severity of CP, but there can be heterogeneity in muscle deficits across individuals and even within the same person across the body (Noble et al., 2014; Finbraten et al., 2015; Peterson et al., 2015; Reid et al., 2015; Handsfield et al., 2016; Whitney et al., 2020e). These altered, and often unpredictable, body size and composition profiles complicates seemingly simple total body (e.g., height, body mass) or regional (e.g., waist:hip ratio, skinfolds) anthropometric measures. As many routinely used clinical measures rely on one or more aspects of anthropometrics, a critical appraisal of what these measures are actually measuring is needed.

### Example #1—Obesity Risk

**Figure 1:**
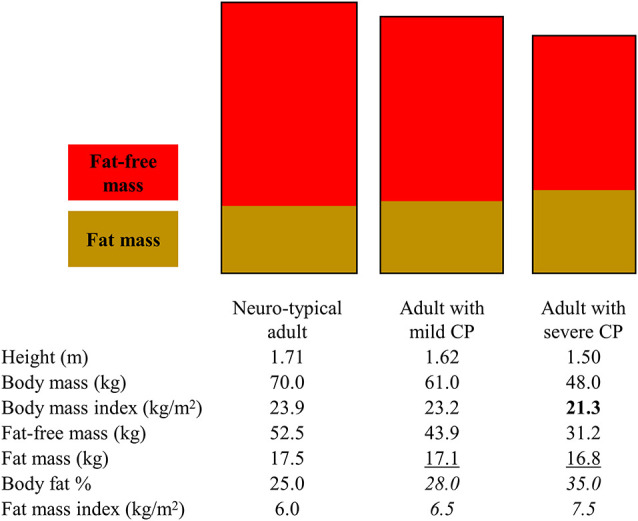
Illustration to highlight how various body fat measures can lead to different interpretations of obesity risk for adults with mild and severe cerebral palsy (CP). The rectangles represent different body dimensions (note the height and width difference) and non-sex-specific fat and fat-free mass distribution. The body composition measures in the table below the rectangles represent non-sex-specific data informed by published studies on adults with and without CP. Bold text represents when a body fat measure may present as low in CP. Underlined text represents when a body fat measure may present as similar in CP. *Italicized test represents when a body fat measure may present as high in CP.* Depending on the body measure analyzed, interpretations may include that adults with CP have higher, similar, or lower obesity risk.

The altered body mass and proportion of fat and fat-free tissue among adults with CP can increase or decrease values of measurements meant to capture obesity risk independent of actual body fat status (Figure 1).

BMI is often used clinically to assess obesity risk even in CP. The underlying assumption is that an increasing BMI above 25 kg/m^2^ for adults is due to increasing body fat. However, BMI is a ratio of total body mass relative to total height and therefore directly reflects on the total body mass rather than fat tissue only: BMI = body mass (kg)/height (m^2^), where body mass = fat mass + fat-free mass. BMI underestimates total body fat and overestimates total body fat-free mass among individuals with CP, with the degree of mis-estimation being greater for more severe forms of CP (Whitney et al., 2019b). This is mainly due to the altered and/or lowered body mass (numerator in BMI ratio) driven by underdeveloped fat-free mass (larger component of body mass), thus lowering the BMI value.

Interpreting values that specifically measure fat mass, as opposed to measures that provide a proxy (e.g., BMI), can also be misleading, but in different ways depending on how fat mass is examined. The total amount of fat mass, derived from a whole body dual-energy x-ray absorptiometry scan for example, may underestimate body fat when compared to normative values as it does not account for the shorter stature in CP. However, caution is advised when examining fat mass relative to other body stature measures. For example, body fat is frequently examined as body fat %, or the proportion of body fat relative to body mass, but this measurement is dependent on fat-free mass: body fat % = fat mass/(fat mass + fat-free mass) × 100. The assumption that the status of and changes in body fat % are due to changes in fat mass does not hold for CP. The lower fat-free mass in CP drives down the denominator, thus raising the body fat % value, which happens independent of fat tissue, leading to the erroneous interpretation that the extent of body fat is higher than it actually is (Whitney et al., 2019b, 2020e). Changes in body fat % over time are driven in part by changes in fat-free mass, which could complicate monitoring obesity risk using any measure of body fat % that is dependent of fat-free mass for adults with CP. Fat mass index has recently been proposed for CP (Whitney et al., 2019b, 2020e): fat mass index = fat mass (kg)/height (m^2^). This may be a preferred method as it accounts for height and is independent of fat-free tissue, thus possibly better capturing body fat relative to the unique body profiles in CP.

However, some fat depots are more deleterious to metabolic health than others. The extent of total body fat may not capture the distribution of fat among various depots in the same way for adults with CP as compared to the general population. There is evidence that fat partitioning among various depots can be unique in CP, which is governed by cellular trafficking of lipids and possible different molecular origins of fat tissue (e.g., fibro-adipogenic precursors), often in response to alterations in function and pathophysiology (Bergouignan et al., 2009; Uezumi et al., 2010; Penton et al., 2013). Studies in children and adults with CP have found higher visceral fat in the abdomen and higher musculoskeletal fat in the lower extremities compared to matched controls, but similar subcutaneous fat between CP and non-CP groups at these same sites (Johnson et al., 2009; Peterson et al., 2015; Whitney et al., 2017, 2020e), and when examined, similar BMI between groups (Johnson et al., 2009; Whitney et al., 2017, 2020e). This calls into question the utility of subcutaneous-derived approaches to assess obesity risk in CP. For example, skinfolds measure the thickness of subcutaneous fat at various regions of the body, which are then used in equations to estimate total body fat, fat-free mass, and body fat %. The underlying assumption is that fat distribution to subcutaneous and other regions are relatively consistent across individuals, thus allowing for inferences on total body fat; but this may not hold for CP. The consequence is under-detection of the extent of fat infiltration in depots that are more deleterious to pathophysiology.

### Example # 2—Bone Health

**Figure 2:**
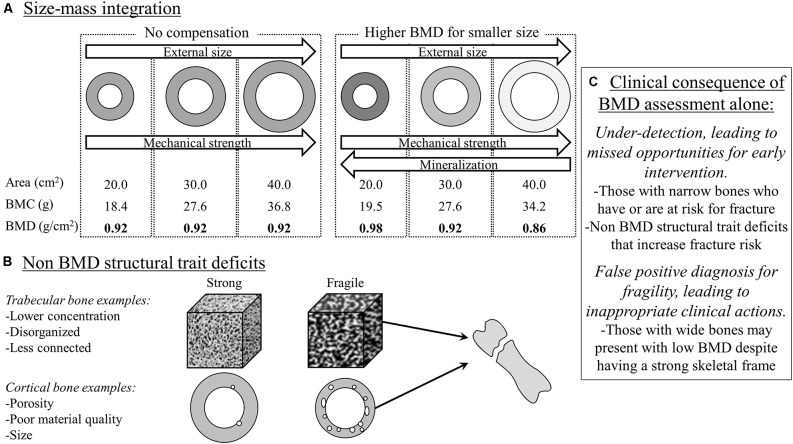
Bone mineral density (BMD), a ratio of bone mineral content (BMC)/bone area, via dual-energy x-ray absorptiometry (DXA) is often used to assess for fracture risk. However, BMD alone can be misleading as it does not provide information on size, which is a major determinant of fracture risk. The “donut” shape represents a cross-sectional diaphysis, with the gray being cortical bone and the open circle being the bone marrow. The white in the cubes represents trabecular bone microarchitecture. **(A)** BMD alone may miss the size-related attributable fracture risk regardless of BMC-area covariation (left panel in **A**), but especially after considering that narrow and wide bones have proportionally greater and lesser mineralization, respectively (right panel in **A**). **(B)** DXA-derived BMD has limited value in situations where BMD may not be reflective of other structural traits in defining fracture risk at the same or other skeletal sites. **(C)** The clinical consequence is under-detection or a false positive diagnosis for bone fragility. In summary, DXA-derived bone traits provide important information to assess fracture risk, but more structural information and at different sites may be needed to better detect and monitor fracture risk for adults with cerebral palsy.

In the clinical setting, bone health is often operationalized as fracture risk. Fracture risk is typically assessed by interpreting the measure, bone mineral density (BMD), derived from dual-energy x-ray absorptiometry. The assumption is that a higher BMD (up to a point before it becomes pathological) indicates a stronger bone that is more resistant to fractures. However, BMD in this context is a ratio: BMD = bone mineral content (BMC)/bone area. The smaller bones in CP due to a narrower outer bone size (e.g., bone area; Whitney et al., 2017, 2022), drives down the denominator, thus raising the BMD ratio value independent of the numerator, bone mass (i.e., BMC), leading to the erroneous interpretation that the bone is stronger than it actually is (Figure 2).

However, there is more to bone than density alone. While BMD reflects on the extent of mineralization relative to that bone’s size, it misses out on other important structural information, some of which is provided by the dual-energy x-ray absorptiometry report, namely bone area. Outer bone size is a major determinant of fracture risk. Using basic biomechanical principles, the ability of a long bone to resist fractures is proportional to the 4th power of the distance from the central axis to the outer bone perimeter in the transverse plane (Ruff and Hayes, 1988). Simply put, whole bone strength is particularly sensitive to outer bone size, but bone size is not part of routine clinical interpretations of fracture risk. In a recent study of adults with CP (Whitney et al., 2022), higher vs. lower BMD was associated with a greater risk of fractures. This seemingly paradoxical association was explained when the individual components of the BMD ratio were examined: bone area was the strongest predictor (out of area, BMD, and BMC) of fracture risk, such that a smaller bone area was associated with a greater fracture risk.

Emerging work in the general population is highlighting the adaptability of the skeleton, which may provide a novel framework to understand how the skeleton in adults with CP is or is not appropriately adapting. There are various biological and structural pathways in which bones are constructed (during growth) and maintained (adult aging), which is governed by the bone’s outer size relative to bone length (Seeman, 2003; Schlecht and Jepsen, 2013; Bolger et al., 2020). In typical aging, smaller diameter bones (relative to bone length) increase the relative extent of mineral deposition (BMC) and cortical area, which increases the extent of mineralization and therefore raises the BMD value. These processes are presumed to be an adaptive mechanism to increase the bone’s stiffness to compensate for the smaller and mechanically weaker skeletal frame. Conversely, wider diameter bones (relative to bone length) exhibit a lower extent of mineralization and proportionally thinner cortices, which lowers the BMD value. These processes are presumed to be a wide bone’s adaptive mechanisms to minimize excess mass given the mechanically stronger, wider skeletal frame. Crudely speaking, given the smaller outer bone size on average among individuals with CP (Whitney et al., 2017, 2022), if the bone is compensating appropriately, we would expect to see BMD values above normative values. However, it is more common to see BMD values well below normative values, suggesting, at least, a double-threat to structural bone fragility: a narrower bone that is insufficiently stiff.

Further research will be needed to determine if fracture risk in CP is better captured by multiple structural traits, and what part of the body may be appropriate; e.g., lower vs. upper extremities or less vs. more involved side for hemiplegic. A first step is to re-consider our over-reliance on the BMD ratio value for adults with CP. While BMD is an important trait to understand whole bone strength, it is one piece of the puzzle for this skeletally complex population.

### Example # 3—Kidney Health

Kidney health is assessed through clinical measures of kidney function. Lower kidney function can reflect different stages of chronic kidney disease based on established thresholds. In the clinical setting, kidney function is assessed by glomerular filtration rate (GFR). The gold standard is to measure GFR (mGFR), but clinical mGFR tests are invasive and time-consuming. Therefore, GFR is almost always estimated (eGFR) using the serum biomarkers, creatinine and/or cystatin c, and equations established from the general population without CP. However, creatinine and cystatin c are regulated in part by muscle and fat tissue, respectively, and are therefore differently impacted by the unique body composition in CP. This can impede accurate assessment of kidney function.

Most pressing is the creatinine-based eGFR, which is more commonly performed clinically and part of routine blood work. Adults with CP have lower levels of creatinine due to low muscle mass, thus augmenting the eGFR value which is exacerbated by greater severity of CP (Whitney et al., 2021b). The eGFR value would suggest that the patient’s kidney function is better than it actually is. The clinical consequence of relying on eGFR for CP is a missed opportunity for early intervention, such as a nephrology referral and preventing or slowing kidney disease progression by, for example, controlling hypertension, mitigating nephrotoxic medication exposure, or proper medication dosing.

To illustrate the clinical consequence of relying on creatinine-based eGFR for CP, we present a novel case study. We begin by highlighting a disparity. Established clinical practice guidelines recommend performing an mGFR test when eGFR is likely to be inaccurate, such as in CP (KDIGO, 2013). Yet, in a recent 20-year period, of the >1,700 adults that had mGFR documented in the Michigan Medicine System, <10 patients had CP (cannot provide exact number for de-identification purposes), which is inconsistent with clinical practice guidelines (KDIGO, 2013). We postulate that this finding is representative across other institutions. One patient was a Caucasian woman in her mid-30s at the time of her mGFR test, GMFCS I (mild severity of CP), and had a competitive running history in High School. Her mGFR test was considered reliable using routine clinical standards (i.e., coefficient of variation across all sampled time periods was 9.4%). Her mGFR was 87.8 ml/min/1.73 m^2^, which is lower (indicating poorer kidney health) than the average from the general population of young adults her age: i.e., 30–39 years of age, 107 ml/min/1.73 m^2^ (Coresh et al., 2003). Her creatinine level was 0.69 mg/dl, which is in the normal range. Using the common clinical equation, CKD-EPI (Chronic Kidney Disease Epidemiology Collaboration), which is recommended when mGFR >60 ml/min/1.73 m^2^, her eGFR was 114.0 ml/min/1.73 m^2^, which overestimated her mGFR by ~30%, but presents clinically as having slightly better kidney function for her age.

While this patient’s mGFR is not suggestive of kidney disease alone, she is young and relatively healthy. Therefore, the 30% discrepancy between eGFR and mGFR may be a conservative estimate for the greater adult population with CP, which may become more disparate with older age and for those with more severe forms of CP. Nevertheless, even a 30% overestimation by eGFR can prevent many adults with CP that have lower eGFR values, but that are still considered “normal” kidney function, from getting the nephrology care and attention they need.

## Discussion

Emerging research is highlighting accelerated health declines as children with CP age into and throughout their adult years, underscoring the importance of preventive care (Day et al., 2007b; van der Slot et al., 2013; Ryan et al., 2014; Himmelmann and Sundh, 2015; Heyn et al., 2019; O’Connell et al., 2019; Smith et al., 2019; Whitney et al., 2019a, 2021a). However, for preventive care, what works for the general population may not work the same for adults with CP. This Perspective showcases that even reliable and repeatable clinical tests must be met with caution when caring for the aging person with CP or other neurodevelopmental disability, considering what the test or measure is actually measuring and the underlying assumptions.

To date, there is not enough empirical evidence to provide sound clinical guidance for which preventive measures to use and what values to interpret for adults with CP. Our perspective is that there may need to be different screening measures (in addition to different value thresholds) to capture health constructs for certain segments of the adult population with CP, such as by severity and/or age. For example, BMI or fat mass index with revised value ranges may capture obesity risk for young adults with mild CP, but other measures, such as clinical imaging of intramuscular fat or waist circumference (Ryan et al., 2014), may be better suited for older adults with CP or those with severe forms of CP at any age. Given the anatomical and physiological complexities of aging with CP, there may also need to be a combination of measures to assess for a single health construct.

Future research is needed to identify how to better utilize the currently available methods to assess for health constructs among adults with CP, or to develop better methods. This is important as accurate assessments of health constructs and developing normative values for adults with CP can help clinicians identify when interventions are needed, when medication dosing should be modified, and guide primary and secondary prevention goals to improve the health and quality of life of the individual with CP.

## Data Availability Statement

The original contributions presented in the study are included in the article, further inquiries can be directed to the corresponding author.

## Author Contributions

DW and EH conceptualized the work. All authors provided intellectual input. DW wrote the first draft. All authors contributed to the article and approved the submitted version.

## Conflict of Interest

The authors declare that the research was conducted in the absence of any commercial or financial relationships that could be construed as a potential conflict of interest.

## Publisher’s Note

All claims expressed in this article are solely those of the authors and do not necessarily represent those of their affiliated organizations, or those of the publisher, the editors and the reviewers. Any product that may be evaluated in this article, or claim that may be made by its manufacturer, is not guaranteed or endorsed by the publisher.
